# The impact of local government debt on urban environmental pollution and its mechanism: Evidence from China

**DOI:** 10.1371/journal.pone.0263796

**Published:** 2022-03-10

**Authors:** Zhenyu Qi, Siying Yang, Dawei Feng, Wenzhi Wang

**Affiliations:** 1 Research Base of Xinjiang Macroeconomic Early Warning System, Xinjiang University, Urumqi, Xinjiang, China; 2 School of Economics and Management, Xinjiang University, Urumqi, Xinjiang, China; 3 Centre for China Public Sector Economy Research, Jilin University, Changchun, Jilin, China; 4 Economics School, Jilin University, Changchun, Jilin, China; 5 Institute of Industrial Economics, Jiangxi University of Finance and Economics, Nanchang, Jiangxi, China; Shenzhen University, CHINA

## Abstract

As an important financial means for governments to improve the quality of economic development, government debt greatly affects the quality of local environmental governance. Based on a theoretical mechanism analysis that uses the pollutant emissions panel data and new caliber urban investment bond data of 273 cities in China, this paper empirically tests the impact of local government debt on urban emission reduction and the mechanism that drives this impact. We find that local government debt significantly promotes urban emissions reduction, and as urban pollution becomes more aggravated, this promoting effect has a dynamic path, first strengthening and then weakening. The role of local government debt in promoting urban emission reduction is characterized by both temporal and spatial heterogeneity. A mechanistic analysis shows that local government debt can promote urban emission reduction by promoting urban environmental innovation, with green invention patents demonstrating a stronger intermediary role than green utility model patents.

## Introduction

As an economic power and a highly populous country, China’s environmental protection issues deserve direct attention. Since the reform and opening up in 1978, China’s economy has achieved rapid growth. However, with this rapid economic development, China’s environmental pollution has also become increasingly severe, especially the deterioration of air quality. According to the *Bulletin of the State of China’s Ecological Environment in 2019* [1], the air quality compliance rate in China’s cities in 2019 was 46.6%, and the proportion of cities with acid rain was 33.3%. Environmental pollution has severely hindered the sustainable development of China’s economy and endangered people’s health. In September 2013, the Chinese government formulated and began implementation of the *Air Pollution Prevention and Control Action Plan* [2]. In October 2017, the Chinese government clearly stated that it would provide more high-quality ecological products, continue to promote the prevention and control of air pollution, and win the battle to defend the blue sky.

Ecological products have the attributes of public goods, so the government should take responsibility for improving the ecological environment and promoting sustainable development. The ability of government to strengthen environmental governance is based on certain fiscal expenditures [3] and so, to some extent, ensuring the fiscal expenditure capacity of local governments has become a prerequisite for government participation in and support of environmental protection. In 1994, China implemented a tax-sharing system reform whereby the central government gained more fiscal revenue, while local governments assumed greater responsibility for fiscal expenditures; this created a dilemma, as local governments’ fiscal revenue capacity did not match their expenditure responsibilities. To alleviate their financial difficulties and improve their expenditure capacity, local governments often engage in debt financing through local investment and financing platforms, allowing them to play both a leading and a supporting role in environmental pollution control. However, the system of political centralization and economic decentralization with Chinese characteristics has also led to a "promotion tournament" among local officials. The local economic growth rate significantly affects the promotion and political status of local officials, and it is easy to distort the financial expenditure incentives of local governments, which leads to a tendency among local governments to focus more on production than environmental protection. Therefore, government debt financing may also lead to a more extensive economic growth mode, thereby aggravating environmental pollution. It is thus clearly necessary to deeply explore the impact of local government debt on environmental pollution in China.

Some studies have examined the relationship between public expenditure and environmental pollution. López et al. (2011) focused on productive pollutants, and their results showed that public expenditure may affect air pollution through scale effects, structure effects, technology effects and demand effects [4]. Halkos and Paizanos (2013) held that public expenditure can influence consumers’ budget, income, and product price through scale effects, regulation, governance quality and the indirect support of special interest groups and thereby influence the emission of consumption-generated pollutants [5]. López and Palacios (2014) used a time-varying country effect model to conduct an empirical study and found that during the period from 1995 to 2008, both total government expenditure and public goods expenditure in many rich EU countries had a negative correlation with production-generated pollution [6]. Hua et al. (2018) studied the relationship between human capital accumulation, R&D, and air pollution by using data from Chinese cities. They found that fiscal expenditures on education and R&D were negatively correlated with SO_2_ emissions [7]. Halkos and Paizanos (2016) also supported this view. They conducted a vector autoregressive analysis of CO_2_ emissions in the United States and found that public expenditures can reduce emissions, especially those expenditures related to environmental protection [8].

The existing literature on government debt mostly focuses on its impact on economic growth [9–11], inflation [12], real interest rates [13] and other economic variables, and less attention has been given to the ecological effects of government debt. Against the background of sustainable development strategies, there remains room to expand the research on local government debt. To the best of our knowledge, only a few studies have analyzed the relationship between government debt and environmental pollution. Fodha and Seegmuller (2014) constructed a generation overlapping model that included consumers, enterprises and government, and found that as government debt increases, the constraint of stabilizing public debt will reduce the proportion of consumer income used for savings and that environmental public policies may lead the economy to fall into an “environmental poverty trap”; thus, government debt is not conducive to reducing environmental pollution [14]. Similarly, Clootens (2017) constructed a two-period OLG model to theoretically analyze the relationship between public debt and environmental pollution. He found that when the debt ratio is below a certain level, public debt has a positive impact on environmental quality, but when the debt ratio exceeds this level, the impact becomes negative [15]. Zhang and Zhao (2018) found that the expansion of government debt creates great pressure for debt repayment. To obtain funds for debt repayment and poverty reduction, local governments may lower environmental standards to attract external investment, which will aggravate environmental pollution [16]. Carratù et al. (2019) used the panel data of 24 European countries from 1996 to 2015 to study the relationship between public debt and air pollution and found an obvious nonlinear relationship between them. The higher the proportion of public debt to GDP is, the weaker the emission reduction effect of public debt [17]. Accordingly, only a few studies investigated the impact of government debt on environmental pollution, and they mainly conducted a theoretical analysis. Several papers used national or provincial panel data for empirical analysis, but the sample size was small, the research results were vulnerable to accidental factors, and the persuasion effect was not sufficiently strong. We did not find any literature with an empirical analysis of the environmental effects of government debt that used large sample data at the city level.

In theory, due to the positive externalities of emission reduction activities and market failures, sustainable economic development is inherently dependent on government forces. Local government behaviors, especially government expenditures, will have an important impact on pollutant emission reduction. Government debt will affect fiscal expenditures on environmental protection, making the marginal private benefits and marginal private costs equal to the marginal social benefits and marginal social costs, thereby internalizing externalities. This is also the theoretical basis for the effect of local government debt on pollutant emission reduction. In practice, China’s local government debt has grown rapidly in recent years; simultaneously, China’s total emissions of conventional air pollutants reached a peak and then gradually decreased. For example, SO_2_ emissions reached an inflection point in 2006 and then declined steadily, while NO_x_ emissions fell for the first time in 2012, and the downward trend has continued since then. Is there an intrinsic relationship between local government debt financing and pollutant emissions? Does local government debt affect urban pollutant emissions? If it does, what is the mechanism through which it operates? What are the dynamic characteristics of the impact of local government debt on urban pollutant emissions? Answering the above questions has important theoretical and practical significance for understanding the logic of local government expenditure behavior, standardizing government debt financing and promoting green development against a background of sustainable development.

Based on the panel data of 273 cities in China from 2006 to 2015, this paper empirically tests the impact of local government debt on pollutant emissions through a theoretical mechanism analysis. In addition, we explore the temporal and spatial heterogeneity of the emission reduction effect of local government debt. Finally, we conduct an in-depth exploration of the mechanism through which local government debt affects urban emission reduction. The innovation of this paper is reflected in the following aspects. First, as mentioned above, the existing research pays little attention to the emission reduction effect of government debt. This paper innovates by taking government debt into account as a factor influencing urban emission reduction, and the emission reduction effect of local government debt and its spatial-temporal heterogeneity are investigated. We found that local government debt significantly promotes the reduction of urban pollutant emissions. Second, considering China’s reform of its administrative management system, the changing characteristics in the scale of government debt and the differences in local economic development stages, this paper further investigates the temporal and spatial heterogeneity of the impact of local government debt on urban emission reduction. The Ministry of Environmental Protection was established in China in 2008, and the scale of local government debt also started to expand rapidly; the results show that since then, local government debt has significantly promoted urban emission reduction, while before 2008, this effect was not significant. Regarding spatial heterogeneity, government debt has no significant impact on urban emission reduction in eastern China but can significantly promote urban emission reduction in central and western China. Third, previous literature neglected to investigate the mechanism through which local government debt impacts urban emission reduction. To affirm the emission reduction effect of local government debt, this paper further investigates this mechanism based on the intermediary effect model and Sobel test. We find that local government debt can promote urban environmental innovation and thereby promote urban emission reduction.

The rest of this paper is arranged as follows: the second section conducts a theoretical analysis of the impact of local government debt on urban pollutant emission reduction and proposes the research hypothesis; the third section introduces the empirical analysis model, variables and data; the fourth section provides the empirical analysis, which investigates the impact of local government debt on urban pollutant emission reduction and its mechanism; and the fifth section includes the main conclusions and this insights of this paper.

## Theoretical basis and research hypothesis

Arguments regarding the effect of government debt on pollutant emissions are based on the externality of the ecological environment and the government’s environmental governance functions. To promote the sustainable development of the economy, local governments that face budget constraints may have to use debt financing to increase environmental protection expenditures and improve the quality of the ecological environment. Therefore, there is a solid theoretical basis and practical possibility for local governments’ debt financing to affect urban pollutant emissions.

On the one hand, government subsidies help to promote the reduction of urban pollutant emissions. By borrowing, governments can offer more financial subsidies for environmental protection. Government subsidies to producers and consumers can guide their behavior and promote emission reduction and the development of a green economy [18]. For producer subsidies, the Chinese government provides subsidies to enterprises that purchase environmentally friendly production equipment and recycle production waste. It also provides financial subsidies representing a certain proportion of project investment for projects that adopt new energy-saving and pollutant emissions-reducing technologies or for relevant enterprises to promote new environmentally friendly products. Subsidies are also granted to enterprises that eliminate or scrap outdated production processes, technologies, and equipment with high pollution emissions in accordance with certain standards, especially in industries with overcapacity. Regarding consumer subsidies, the government often grants subsidies to consumers who buy energy-saving, low emission, and clean production products. For example, in some areas, the government provides purchase subsidies based on the mileage of new energy vehicles. Financial subsidies can obviously promote the sales of new energy vehicles and effectively promote a reduction in urban pollutant emissions [19]. The government also provides incentive subsidies to consumers to eliminate old vehicles with high pollution emissions.

On the other hand, the government can support environmental protection through green investment [20]. The government often invests through debt financing to establish more efficient and energy-saving public transportation systems and enterprises with higher standards for clean production or for energy efficiency, which can help to reduce pollutant emissions and achieve sustainable development goals [21]. For example, the government will raise funds by issuing urban investment bonds to improve infrastructure such as roads and railways, to improve travel efficiency and to reduce emissions from traffic. For another example, in winter, many residents in northern China use stoves, fire walls, small boilers and other independent heating methods that present the disadvantages of low energy efficiency, high pollution, and high emissions. To improve environmental quality, local governments have invested in the construction of heating enterprises to achieve central heating, significantly reducing pollutant emissions. In recent years, the Chinese government has shut down many small coal-fired power plants with high coal consumption and high pollutant emissions, implemented the ultralow emission project for coal-fired power plants, and built many new power plants with an average coal consumption of less than 300 grams per kilowatt hour. These green investments tend to be massive in scale, and one of the important sources of funds is the government investment and financing platform. To summarize the analysis, this paper proposes the following research hypothesis.

**Hypothesis 1**: Local government debt can effectively promote the reduction of urban pollutant emissions.

A good ecological environment is a type of important public good [22]. As it is nonexclusive and noncompetitive in the process of consumption, there is always serious market failure in its supply. Therefore, the effective supply of this type of public good is an important function of government. With the development of the economy, this function is becoming increasingly more important, because a good ecological environment is an important condition for realizing sustainable economic development [23–25]. Before China’s 11th Five Year Plan (2006–2010), the environmental protection objectives of several five-year plans were not achieved. In particular, the 10th Five Year Plan (2001–2005) stipulated that SO_2_ emissions and COD would be reduced by 10%, but in fact, SO_2_ emissions increased by 27.8%, and COD decreased by only 2.1% [26]. In consequence, the Chinese government began to fully understand the arduous work involved in environmental protection and devoted more attention to it, increasing its investment in environmental protection since the 11th Five Year Plan. In March 2008, China established the Ministry of Environmental Protection to strengthen the control of environmental pollution. Since then, the Ministry of Environmental Protection has formulated and issued emission standards for air or water pollutants in many industries, strengthened national environmental quality monitoring, and promoted the formulation of many environmental protection policies. The central government also began to treat local government leaders as the first person responsible for environmental protection, included environmental protection into the assessment of local government leadership, and regarded it as an important basis for the selection, reward, and punishment of officials. In December 2011, the Seventh National Conference on Environmental Protection was held, and the State Council signed a responsibility statement for the pollution reduction targets in the 12th Five Year Plan period with provincial leaders. Therefore, after the establishment of the Ministry of Environmental Protection of China, the local government’s environmental protection function was enhanced, and because local government debt financing revenue is usually used more for environmental protection, the promotion effect of local government debt on urban pollutant emission reduction was also enhanced. To summarize the analysis, this paper proposes the following research hypothesis.

**Hypothesis 2**: Local government debt is characterized by temporal heterogeneity in promoting the reduction of urban pollutant emissions. After the establishment of the Ministry of Environmental Protection, local government debt played a stronger role in promoting urban emission reduction.

Compared with the eastern region, the central and western regions of China have a lower level of economic development but are rich in natural resources. The reserves of coal, oil and natural gas resources in Xinjiang, Shaanxi, Inner Mongolia, and Shanxi account for a high proportion of the total reserves in China, and these provinces serve as China’s energy base. Heavy industries related to the development and utilization of mineral resources, such as coal mining, oil and gas processing and nonferrous metal metallurgy, are the main industries in central and western China. The rapid development of these high pollutant emission industries has led to serious damage to the ecological environment in central and western China. In addition, due to the shallow soil layer, high altitude, water shortages and other reasons, once the ecological environment in central and western China is destroyed, it will have difficulty recovering. Therefore, the central and western regions of China urgently need their local governments to increase environmental protection expenditures. Government debt financing can greatly alleviate the pressure created by government environmental protection expenditures and improve the ecological environment of the central and western regions. In contrast, cities in eastern China have a better economic development level and eco-environmental quality than those in the central and western regions, their sustainable development is at the forefront for China, and their ecological environmental governance is less dependent on the government. Consumers in eastern China have a strong sense of environmental protection and are even willing to pay extra money for the purchase of green products [27–29], and cleaner production has reached a very high level. Compared with the central and western regions, the eastern region has more local government debt and environmental protection expenditure. According to the law of diminishing marginal returns, the impact of local government debt on urban pollutant emission reduction in eastern China may be relatively small or even not significant. To summarize the analysis, this paper proposes the following research hypothesis.

**Hypothesis 3**: Local government debt exhibits spatial heterogeneity in promoting the reduction of urban pollutant emissions, and the promotion effect on urban emission reduction in the central and western regions is stronger than that on the eastern regions.

Local government debt has a significant impact on technological innovation and may further affect environmental pollution. Environmental innovation seeks to reduce environmental damage in the process of economic development [30], can promote energy conservation and emission reduction, and has strong positive externalities. However, green technology usually has a long development cycle and is characterized by high cost, high risk and low profit [31, 32]; new products adopting green technology often have higher costs and lack marketing channels. Therefore, enterprises lack the intrinsic motivation to engage in environmental innovation activities and need the support and guidance of government [33–36]. In the context of green development strategy, debt financing can help increase the government’s financial support for green technology research and development projects, give full play to the leverage capability of financial funds, and guide more enterprises to engage in environmental innovation activities with both economic and ecological benefits. At the same time, local government debt reduces taxes in the short term, which will reduce the tax burden of enterprises and produce the "tax depression" effect [37]. Therefore, local government debt can stimulate private investment, ensure the supply of funding for innovation activities, and have a significant promoting effect on urban environmental innovation. In addition, local government debt can also reduce the crowding out effect of government productive expenditure on its science and technology expenditure, increase the proportion of science and technology expenditure in total government expenditure, and thereby promote the level of urban environmental innovation. Under the Chinese-style decentralized fiscal system [38, 39], the local economic scale and growth rate are the key criteria for evaluating the performance of local officials. As a result, local officials tend to direct fiscal spending toward areas that can expand the economy in the short term [40, 41]. Faced with budget constraints, local government officials tend to reduce science and technology expenditure to invest more in productive fields, which leads to productive fiscal expenditure crowding out government expenditure directly related to scientific and technological innovation. To some extent, local government debt alleviates local government budget constraints, reduces the "crowding out effect" of productive expenditure on fiscal science and technology expenditure and is conducive to ensuring the scale of government science and technology expenditure and promoting the level of urban environmental innovation. Therefore, government debt is favorable to increasing the investment of environmental innovation and improving its benefits [42–44], and then reducing the emission of urban pollutants. To summarize the analysis, this paper proposes the following research hypothesis.

**Hypothesis 4**: Local government debt can effectively promote the level of urban environmental innovation and thereby promote the reduction of urban pollutant emissions.

## Research design

### Model

This paper focuses on the impact of local government debt on the reduction of urban pollutant emissions and its mechanism. First, a regression model, as shown in Eq (1), is established to examine the effect of government debt on pollutant emissions to test whether hypothesis 1 is valid.

$${pollution}_{it}=\alpha_{0}+\alpha_{1}{debt}_{it}+\sum \alpha_{j}X_{jit}+u_{i}+v_{t}+\varepsilon_{it}$$ (1)

where *pollution*_*it*_ represents the pollutant emissions of city *i* in year *t*. This paper takes urban SO_2_ emissions as an example. *debt* represents the amount of local government debt, and its regression coefficient reflects the impact of government debt on urban SO_2_ emissions, that is, whether government debt promotes urban emissions reduction. *X*_*j*_ represents a collection of control variables to control other economic and social factors that affect urban pollutant emissions. *u*_*i*_ and *v*_*t*_ represent time and individual dummy variables, respectively. *ε*_*it*_ is the random error term.

In addition, Clootens (2017) and Carratù et al. (2019) show that the impact of government debt on environmental pollution is nonlinear [15, 17]. Therefore, this paper adds the square of local government debt to Eq (1) to test whether the impact of local government debt on urban pollutant emissions is nonlinear, as shown in Eq (2), where *debt*^*2*^_*it*_ represents the square of government debt.


$${pollution}_{it}=\alpha_{0}+\alpha_{1}{debt}_{it}+\alpha_{2}{debt}_{it}^{2}+\sum \alpha_{j}X_{jit}+u_{i}+v_{t}+\varepsilon_{it}$$ (2)

To further examine the dynamic characteristics of the impact of local government debt on urban emission reduction as the degree of urban pollution increases, this paper establishes a quantile regression model, as shown in Eq (3), where *q* represents the quantile, *pollution*_*q*_ represents the degree of urban pollution corresponding to quantile *q*, and *α*_*q*,*1*_ represents the marginal effect of local government debt on urban emissions when the urban pollution degree is *pollution*_*q*_.


$${pollution}_{q,it}=\alpha_{q,0}+\alpha_{q,1}{debt}_{q,it}+\sum \alpha_{j}X_{jit}+u_{q,i}+v_{q,t}+\varepsilon_{q,it}$$ (3)

Finally, to investigate whether local government debt will affect urban emission reduction by affecting the level of urban environmental innovation, this paper further establishes the regression model shown in Eqs (4) and (5), which together with Eq (1), form a complete mediation effect model.


$${e\_inno}_{it}=\beta_{0}+\beta_{1}{debt}_{it}+\sum \alpha_{j}X_{jit}+u_{i}+v_{t}+\varepsilon_{it}$$ (4)

$${pollution}_{it}=\gamma_{0}+\gamma_{1}{debt}_{it}+\gamma_{2}{e\_inno}_{it}+\sum \alpha_{j}X_{jit}+u_{i}+v_{t}+\varepsilon_{it}$$ (5)

In Eqs (4) and (5), *e_inno*_*it*_ represents the level of urban environmental innovation, *β*_*1*_ represents the impact of local government debt on urban environmental innovation, and *γ*_*2*_ represents the impact of environmental innovation on urban emission reduction. If local government debt can affect urban emission reduction, that is, if *α*_*1*_ is significant in Eq (1), then we will further estimate Eqs (4) and (5). If *β*_*1*_ in Eq (4) and *γ*_*2*_ in Eq (5) are both significant, then local government debt affects urban emission reduction by influencing the environmental innovation level, and the intermediary effect is *β*_*1*_*γ*_*2*_. If *γ*_*1*_ is still significant, then the level of urban environmental innovation is a partial mediating variable. Local government debt will not only affect urban emission reduction by influencing environmental innovation but also directly affect it or indirectly affect it through other mechanisms. If *γ*_*1*_ is not significant, the level of urban environmental innovation is a complete mediating variable, local government debt affects urban emission reduction only by affecting the level of environmental innovation, and there is no direct or other indirect mechanism.

### Variables

Based on urban panel data, this paper investigates the emission reduction effect of local government debt, so urban pollutant emissions are the explained variable. The existing literature mostly examines emission reduction effects from two perspectives, namely, exhaust gas and wastewater discharge. This paper follows this approach and constructs an urban exhaust emission index based on urban SO_2_ emissions in the baseline regression analysis and an urban emission reduction index based on wastewater discharge (*pollution_water*) in the robustness test. Considering that secondary industry is the main source of SO_2_ emissions, this paper uses the ratio of urban SO_2_ emissions to the added value of secondary industry as a relative index (*pollution*) to measure urban SO_2_ emissions and uses the absolute value of SO_2_ emissions (*pollution_1*) and urban SO_2_ emissions per capita (*pollution_2*) as alternative variables to test the effect of government debt on urban emission reduction. To reduce the impact of data fluctuations on the empirical results, the above indicators are logarithmically processed.

Local government debt is the core explanatory variable of this paper. To alleviate the dilemma of a mismatch between fiscal revenue and expenditure, local governments often raise funds in the form of urban investment bonds to avoid legal constraints on local government bond issuance. Therefore, urban investment bonds are also called "quasi-municipal bonds", and they account for a high proportion of local government debt. The existing literature mostly uses urban investment bonds to measure local government debt, but existing databases have many defects in sorting the data of urban investment bonds. Therefore, Cao et al. (2019) scientifically established a list of local financing platforms, searched the bond issuance information based on this new list, and matched the bond issuance information to each city, thereby obtaining the number and scale of local government bonds issued [45]. Based on these data, this paper uses the logarithm of the local urban investment bond amount plus 1 as the measurement index of the scale of local government debt (*debt*). Similarly, this paper draws on the practice of Cao et al. (2019) and uses the amount of bonds issued after applying an inverse hyperbolic sine transformation as an alternative variable of the scale of local government debt (*debt_IHS*) to test robustness.

Environmental innovation is the mediating variable in this paper. Kesidou and Wu (2020) measured the level of ecological innovation in each province based on the number of green technology patents obtained by enterprises [46]. This paper draws on this practice and uses the ratio of the number of green patent applications within a city to the population as the index for measuring the level of ecological innovation (*e_inno*) in a city.

In addition, this paper also selects some indicators as control variables affecting urban pollutant emissions, such as the degree of openness (*fdi*), financial development level (*finance*), urban entrepreneurship activity (*entrepreneurship*), resource abundance (*resource*), industrial structure (*industry*), investment scale (*investment*), population density (*population*), economic growth-related stress of local officials (*stress*) and government scale (*g_scale*). The selection and definition of control variables are shown in Table 1.

**Table 1:** Control variable definitions.

Variable name	Variable definition
*fdi*	The proportion of foreign direct investment actually used by cities in GDP
*finance*	The proportion of the deposit and loan balance of local financial institutions in GDP
*entrepreneurship*	The proportion of local private enterprise employees in the total number of employees
*resource*	The proportion of urban mining industry employees in the total urban employment
*industry*	The proportion of added value of nonagricultural industries in GDP
*investment*	The proportion of fixed asset investment in GDP
*population*	The ratio of the urban population to the urban area
*stress*	The ratio of per capita GDP of the city ranked one higher in the same province to the per capita GDP of the focal city
*g_scale*	The proportion of government general budget expenditure in GDP

### Data

The sample is panel data of 273 cities in China from 2006 to 2015. The data of local urban investment bonds were collected by Professor Mao Jie’s team from the University of International Business and Economics, which are disclosed on the website of the editorial department of *Finance and Trade Economics*. For green patent data, we searched the Chinese patent application data on the website of the State Intellectual Property Office, removed non-green patent data according to the international patent classification code, and then counted the number of patents in each city to obtain the urban green patent data. The rest of the data come from the EPS data platform. The statistical characteristics of each variable are shown in Table 2, where the last column of the table shows the correlation coefficient between each variable and urban pollutant emissions. The correlation coefficient between the scale of government debt and urban pollutant emissions is negative at the 1% significance level; this may be because local government debt promotes urban emission reduction. In addition, the correlation coefficient between environmental innovation and urban pollutant emissions is also negative at the 1% significance level, which may be because environmental innovation can effectively inhibit urban pollutant emissions. Of course, the above conjectures still need to be further tested.

**Table 2:** Statistical characteristics of the variables.

Variable	Sample size	Mean value	Standard deviation	Minimum value	Maximum value	Correlation coefficient
*pollution*	2,826	-4.7527	1.0544	-12.9664	-1.1054	1
*pollution_1*	2,827	10.6143	1.0371	0.6931	13.4341	0.4914*
*pollution_2*	2,827	13.9631	1.0801	5.8328	17.1917	0.6355*
*pollution_water*	2,831	-6.9349	0.8803	-10.3289	-2.6152	0.5006*
*debt*	2,760	1.1728	1.6218	0.0000	6.6564	-0.3908*
*debt_IHS*	2,760	1.6524	3.2435	-0.6931	12.6197	-0.3908*
*e_inno*	2,850	0.2061	0.5922	0.0000	8.6453	-0.3802*
*fdi*	2,850	0.0292	0.0292	0.0000	0.1945	-0.2664*
*finance*	2,850	2.0128	0.9574	0.5600	8.7774	-0.2141*
*entrepreneurship*	2,850	0.1061	0.1218	0.0000	1.5243	-0.3828*
*resource*	2,672	0.0078	0.0187	0.0000	0.2390	0.1352*
*industry*	2,849	1.3808	0.6642	0.1006	17.6470	-0.1877*
*investment*	2,849	0.6777	0.2574	0.0872	2.1691	-0.0968*
*population*	2,850	0.0427	0.0325	0.0005	0.2648	-0.3718*
*stress*	2,738	1.1589	0.2405	1.0000	3.3971	0.1212*
*g_scale*	2,850	0.1666	0.0955	0.0426	1.4852	0.0990*

Note:

* indicates a significance level of 1%.

## Empirical results and analysis

### Benchmark regression

First, we estimate the regression model shown in formula (1) based on urban panel data, and the results are shown in Table 3. Regression (1) takes the sulfur dioxide emissions per unit output value of secondary industry as the explained variable for estimation. The results show that the estimation coefficient of the government debt scale is negative at the 1% significance level, which indicates that local government borrowing significantly reduces urban sulfur dioxide emissions. Regression (2) takes the logarithmic sulfur dioxide emissions (*pollution_1*) as the explanatory variable, and regression (3) takes the logarithmic value of per capita sulfur dioxide emissions (*pollution_2*) as the explanatory variable. The results all show that the estimation coefficient of the government debt scale is negative at the 5% significance level. The above results show that local government borrowing has an emission reduction effect, and the larger the scale of government debt is, the lower the emission of sulfur dioxide in cities. Therefore, hypothesis 1 is confirmed.

**Table 3:** Benchmark regressions.

VARIABLES	(1)	(2)	(3)	(4)	(5)	(6)
	*pollution*	*pollution_1*	*pollution_2*	*pollution*	*pollution_1*	*pollution_2*
*debt*	-0.0238***	-0.0171**	-0.0188**	0.0128	0.0101	0.0125
	(0.0085)	(0.0081)	(0.0081)	(0.0169)	(0.0161)	(0.0161)
*debt* ^*2*^				-0.0106**	-0.0079*	-0.0091**
				(0.0043)	(0.0041)	(0.0041)
control variables	Y	Y	Y	Y	Y	Y
time-fixed effect	Y	Y	Y	Y	Y	Y
individual-fixed effect	Y	Y	Y	Y	Y	Y
*constant*	-3.4739***	11.3419***	14.9574***	-3.5254***	11.3033***	14.9132***
	(0.1704)	(0.1623)	(0.1625)	(0.1715)	(0.1633)	(0.1636)
Observations	2,537	2,538	2,538	2,537	2,538	2,538
R-squared	0.6100	0.0545	0.0780	0.6111	0.0561	0.0801

Note:

*** p<0.01,

** p<0.05,

* p<0.1, Standard errors in parentheses.

To further analyze whether the influence of government borrowing on sulfur dioxide emissions has nonlinear characteristics, this paper estimates the regression model shown in formula (2), and the results are shown in regressions (4) to (6) in Table 3. Among them, the square term estimation coefficient of the government debt scale passes the significance test in the three regressions, and all of the coefficients are negative. However, the estimation coefficient of the government debt scale does not pass the significance test. Because the scale of government debt is nonnegative, it can be judged from regression (4) to regression (6) that local government borrowing will significantly promote urban emission reduction, which also confirms hypothesis 1.

The above regression results describe the average marginal effect of local government borrowing on urban emission reduction, but it is difficult to describe the difference in the marginal effect of local government borrowing on urban emission reduction considering cities with different pollution levels. Therefore, we further introduce a quantile regression model to investigate the dynamic trajectory of the impact of local government borrowing on urban emission reduction. Specifically, this paper selects five quantiles (10%, 25%, 50%, 75% and 90%) and takes the averaged sulfur dioxide emissions of secondary industry as the interpreted variable to estimate the regression model shown in formula (3). The results are shown in Table 4. At the 10% quantile, although the regression coefficient of local government borrowing to sulfur dioxide emissions is negative, it is not significant. At the 25%, 50%, 75% and 90% quantiles, the estimation coefficients of local government borrowing on sulfur dioxide emissions are significantly negative. The above results show that when the degree of urban pollution is very low, the emission reduction effect of local government borrowing is not significant, but with the increase in urban air pollution, local government borrowing begins to significantly promote urban emission reduction. Comparing the absolute values of the regression coefficients at different quantiles, it is found that when the quantile is less than 50%, the absolute value of the regression coefficient of local government borrowing increases continuously, from 0.0066 at the 10% quantile to 0.0139 at the 25% quantile and then to 0.0186 at the 50% quantile. When the quantile is higher than 50%, the regression coefficient of the government debt scale decreases with the increase in the quantile, that is, from 0.0186 at the 50% quantile to 0.0134 at the 75% quantile, and further to 0.0119 at the 90% quantile. Therefore, we preliminarily judge that with improvement in the urban innovation level, the promotion effect of local government borrowing on urban emission reduction presents a dynamic change feature and first strengthens and then weakens.

**Table 4:** Quantile regression results.

quantiles	(1)	(2)	(3)	(4)	(5)
	0.1	0.25	0.5	0.75	0.9
*debt*	-0.0066	-0.0139**	-0.0186**	-0.0134***	-0.0119***
	(0.0055)	(0.0059)	(0.0073)	(0.0051)	(0.0044)
control variables	Y	Y	Y	Y	Y
time-fixed effect	Y	Y	Y	Y	Y
individual-fixed effect	Y	Y	Y	Y	Y
*constant*	-4.3879***	-4.3836***	-4.1889***	-3.8929***	-4.1225***
	(0.4334)	(0.6510)	(0.7120)	(0.7289)	(0.6517)
Observations	2537				
R-squared	0.7418	0.7156	0.6936	0.7399	0.7776

Note:

*** p<0.01,

** p<0.05,

* p<0.1, Standard errors in parentheses.

**Fig 1:**
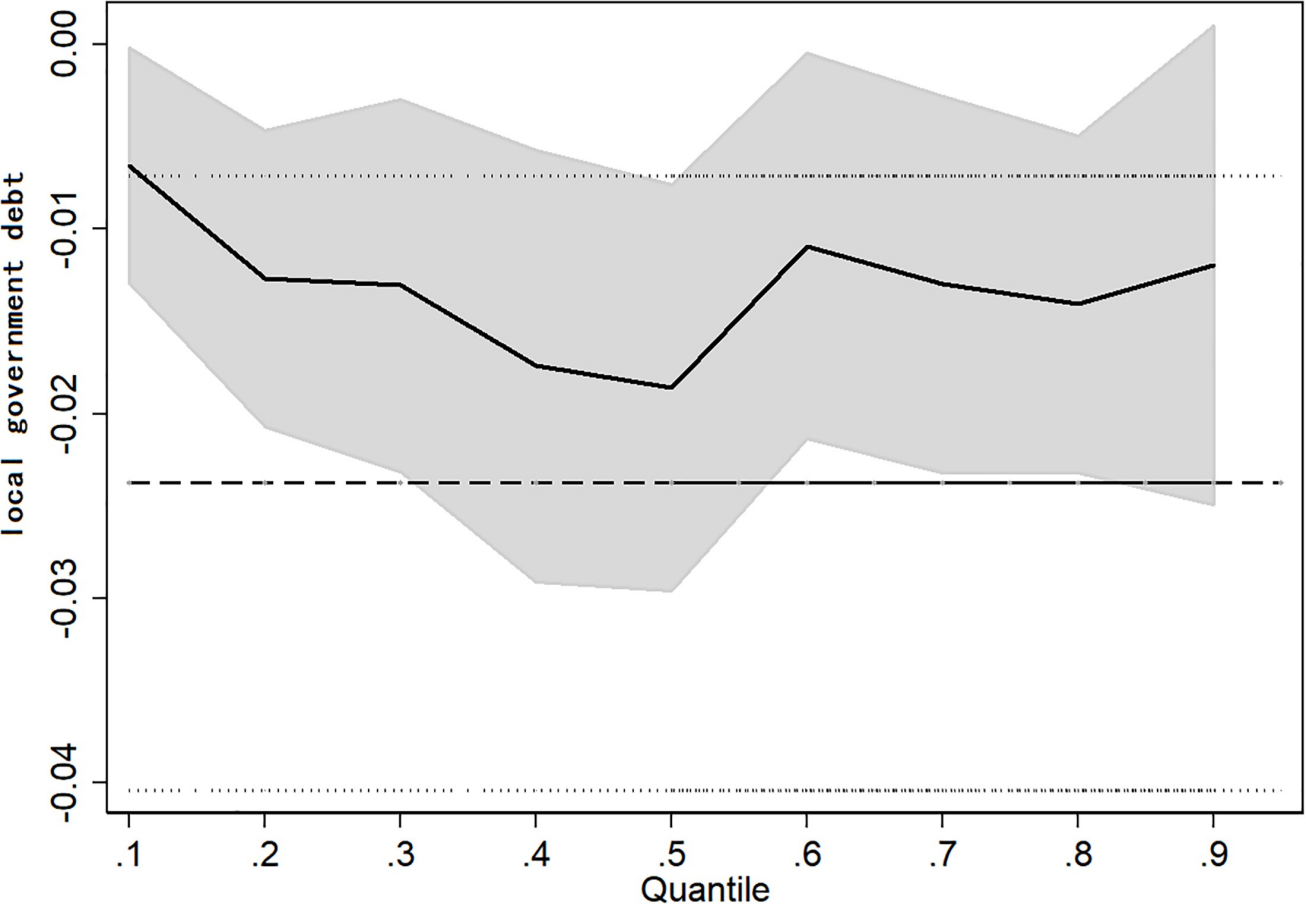
Quantile regression diagram.

To further depict accurately the dynamic change characteristics of the impact of local government debt on urban emission reduction at different quantiles, we present the regression coefficient diagrams of local government debt scales at different quantiles, as shown in Fig 1. With the increase in quantiles, the absolute regression coefficient of local government debt on urban sulfur dioxide emissions is characterized by dynamic change, first increasing and then decreasing overall, which is consistent with the above regression results. This may be because local governments of cities with less pollution experience less pressure and lower costs to reduce emissions and do not need to relieve the pressure of environmental expenditure through borrowing or other means. With an increase in urban pollution, the pressure on local governments to protect the environment gradually increases. Environmental protection expenditure within the government budget cannot meet the requirements of urban emission reduction, so the government needs to alleviate the pressure of fiscal expenditure by accessing debt to promote urban emission reduction. However, in cities with higher pollution, the local ecological environment is relatively fragile. Due to path dependence and for other reasons, the original development pattern of high energy consumption and high pollution has continued in China. Although local government borrowing can promote urban emission reduction to a certain extent, the effect is evidently weakened because the original extensive development model takes some time to change.

### Robustness test

1. Robustness Test I: Replace the method for measuring local government debt. As mentioned above, the amount of bonds issued by the local government after an inverse hyperbolic sine transformation was used as the substitution variable in the robustness test of the scale of local government debt. The results are shown in regression (1) in Table 5. The regression coefficient of the replaced government debt scale is still negative at the 1% significance level, which indicates that local government borrowing significantly promotes urban emission reduction. The above conclusion is robust.

**Table 5:** Robustness test.

	(1)	(2)	(3)	(4)	(5)
	Alternative measurements		Adjust the sample	Address endogeneity problems	
	*pollution*	*pollution_water*	*pollution*	*pollution*	*pollution*
*debt*		-0.0176**	-0.0150*	-0.7029***	-0.4164***
		(0.0084)	(0.0090)	(0.1181)	(0.1398)
*debt_IHS*	-0.0119***				
	(0.0042)				
control variables	Y	Y	Y	Y	Y
time-fixed effect	Y	Y	Y	Y	Y
individual-fixed effect	Y	Y	Y	Y	Y
*constant*	-3.4821***	-6.6278***	-3.2761***		
	(0.1706)	(0.1692)	(0.1866)		
instrumental variable				*debt*_*t-1*_	growth target
Anderson				36.957	13.778
LM Stats				[0.0000]	[0.0002]
Cragg-Donald				37.466	13.802
Wald F Stats				{16.38}	{16.38}
Observations	2,537	2,541	2,303	2,261	2,521
R-squared	0.6100	0.5602	0.6146	0.9905	0.1781

Note:

*** p<0.01,

** p<0.05,

* p<0.1, Standard errors in parentheses.

2. Robustness test II: Replace the urban pollutant indicators. We further use urban wastewater discharge as the measurement index for urban pollutant discharge, specifically, the logarithm of the proportion of urban wastewater discharge, and the added value of secondary industry as the explained variable, and we put these into Eq (1) for estimation. The results are shown in regression (2) in Table 5. The regression coefficient of the local government debt scale is negative at the 5% significance level, which suggests that local government borrowing can promote urban emission reduction, which is consistent with the above conclusion.

3. Robustness Test III: Adjust the sample. China has more than 300 cities, including four municipalities directly under the central government, five cities specifically designated in the state plan, and 27 provincial capitals. We define the municipalities directly under the central government, cities specifically designated in the state plan, and provincial capitals as central cities. Compared with most ordinary cities, central cities play a particularly important role in regional economic development, as they have a relatively high level of economic development and their local governments have strong financing capacity. In addition, central cities often have a better ecological environment. In view of the particularity of central cities in terms of the economy and the environment, we eliminated them from the samples and estimated Eq (1) based on the remaining samples. The results are shown in regression (3) in Table 5. It can be seen that the estimated coefficient of the government debt scale is negative at the 10% significance level, which confirms the above conclusion.

4. Robustness test IV: Address endogeneity problems. Promoting urban emission reduction is a complex and systematic project, as there are many factors affecting urban pollutant discharge, including some non-observable factors. It is difficult to include all the factors affecting urban emission reduction in the model set, and variables will inevitably be omitted in the model setting. In addition, factors such as measurement error and reverse causality for urban pollutant emissions or government debt scale can also lead to endogeneity problems. The instrumental variable method is the most commonly used approach to solve endogeneity problems. Generally, local governments have certain behavioral inertia, as does their borrowing behavior. There is a strong correlation between local government borrowing behavior in the previous year and that in the current period, but the urban ecological environment in the current period would not affect the local government borrowing behavior in the previous year. Therefore, it is an ideal instrumental variable to meet the exogeneity requirements. In this paper, the scale of government debt in the last period was taken as an instrumental variable, and the two-stage least squares method was used to estimate it. The results are shown in regression (4). It can be seen that the regression coefficient of the government debt scale is still negative at the 1% significance level, and there is no problem of unrecognizable or weak instrumental variables in the model. After controlling the endogeneity problem of the model, the emission reduction effect of local government borrowing is still significant.

Under the promotion tournament mechanism, local governments tend to cater to the growth preference of higher-level governments, blindly pursue the expansion of the local economic scale, and show a strong desire to increase fiscal expenditure and intervene in the economy. When there is an imbalance between fiscal expenditure power and responsibility, local governments usually relieve the pressure on government expenditure by borrowing money. Therefore, the growth target of local governments affects their borrowing behavior to a great extent. As mentioned above, the promotion mode of officials in China’s centralized political system leads local governments to adopt a responsible behavior mode, that is, to cater to the economic growth preference of higher-level governments. Thus, there is a strong correlation between the economic growth target of provincial governments and the borrowing scale of city governments, but there is no direct impact on the emission of urban pollutants. Therefore, the growth targets of provincial governments meet the exogeneity requirements and are estimated based on the two-stage least squares method as an instrumental variable. The results are shown in regression (5). The results of the LM test and Wald test show that the model does not have the problem of unrecognized and weak instrumental variables. The regression coefficient of the local government debt scale is still negative at the 1% significance level, which indicates that local government borrowing promotes urban emission reduction and is consistent with the above conclusion.

### Analysis of temporal and spatial heterogeneity

As pointed out in hypothesis 2 and hypothesis 3, the impact of local government borrowing on urban emission reduction may vary between different time periods and different regions. For this reason, this paper adopts the sample regression method to test the above two hypotheses. First, the scale of urban investment bonds issued by local governments through investment and financing platforms has expanded rapidly since 2009. For example, the debt scale in the first eight months of 2009 exceeded 50% of the debt scale in the four years from 2005 to 2008. Second, the Ministry of Ecology and Environment was established in 2008 to further clarify the government’s functional positioning, plans and measures for environmental protection, which effectively enhanced the government’s important role in environmental protection. Therefore, taking 2008 as the cutoff point, this paper subdivides the samples into two subsamples from 2006 to 2008 and 2009 to 2015 and estimates them. The results correspond to regression (1) and regression (2) in Table 6. In regression (1), the estimated coefficient of the government debt scale on urban sulfur dioxide emissions, although negative, is not significant, while in regression (2), the coefficient is negative at the 5% significance level. The above results show that local government borrowing before 2008 has no significant impact on urban emission reduction, but after 2008, local government borrowing significantly restrains urban pollutant emissions, and hypothesis 2 is confirmed. This may be due to the abovementioned reasons: the establishment of the Ministry of Ecology and Environment strengthened the environmental protection function of the government, and local government debt was conducive to the effective implementation of this environmental protection function. Furthermore, the scale of local government debt has expanded rapidly since 2008, thereby giving full play to the scale effect of government debt in promoting environmental governance and restraining urban pollutant emissions. Before 2008, due to the relatively small scale of government debt, although it had a certain promoting effect on urban emission reduction, this effect was small.

**Table 6:** Analysis of temporal and spatial heterogeneity.

	(1)	(2)	(3)	(4)
	Temporal heterogeneity		Spatial heterogeneity	
	Before 2008	After 2008	Eastern region	Central and western regions
*debt*	-0.0220	-0.0182**	0.0027	-0.0347***
	(0.0202)	(0.0090)	(0.0106)	(0.0118)
control variables	Y	Y	Y	Y
time-fixed effect	Y	Y	Y	Y
individual-fixed effect	Y	Y	Y	Y
*constant*	-3.855***	-3.853***	-3.9441***	-3.6722***
	(0.594)	(0.228)	(0.3163)	(0.2144)
Fisher’s Permutation test (P-value)	0.0410		0.0030	
Observations	775	1,762	886	1,651
R-squared	0.474	0.393	0.7382	0.5776

Note:

*** p<0.01,

** p<0.05,

* p<0.1, Standard errors in parentheses.

Economic development in the central and western regions is excessively dependent on the high consumption of energy resources, which leads to an extremely fragile ecological environment. However, the overall level of economic development in the eastern region is relatively high, its pace of industrial transformation and upgrading has been rapid, and it experiences much less environmental pressure than the central and western regions. Such differentiated characteristics of the economic development stage and ecological environmental pressure may lead to differentiated effects of local government borrowing on urban emission reduction. For this reason, we subdivide the samples into two subsamples, namely, one from the eastern region and one from the central and western regions, and estimate them based on Eq (1). The results are shown in Table 6, regression (3) and regression (4). Regression (3) gives the sample estimation results for the eastern region, and regression (4) gives the sample estimation results for the central and western regions. It can be seen that the impact of local government borrowing on urban emission reduction is not significant in the eastern region, but it significantly promotes urban emission reduction in the central and western regions; therefore, hypothesis 3 is confirmed. This may be because most cities in the eastern region have realized their transformation from the traditional extensive development model to an intensive development model, with relatively small environmental costs for economic growth and weak dependence on the government for ecological protection. At this stage, the impact of government borrowing on urban emission reduction will be relatively weak. For the central and western regions, the ecological environment is fragile, and their economic development is relatively lagging. While the government commits to promoting economic growth, it should also strive to improve the fragile ecological environment. When faced with strong budget constraints, local governments will use debt and other means to promote economic growth and ecological protection. Therefore, the impact of local government borrowing on urban emission reduction is not significant in the eastern region but can significantly promote urban emission reduction in the central and western regions.

### Analysis of mediation mechanism

Environmental innovation can guarantee economic benefits while simultaneously improving the ecological environment. Therefore, it is important that the government promote environmental innovation to promote environmental governance. Can government borrowing promote urban emission reduction by promoting environmental innovation? To test whether a mediation effect of environmental innovation exists, this paper further estimates the mediation effect model shown in Eqs (1), (4) and (5), and the results are shown in Table 7. Regression (1) to regression (3) provide the estimation result of the number of green invention patent applications per capita as a proxy variable of urban environmental innovation. Consistent with the above conclusions, in regression (1), local government borrowing significantly inhibited urban sulfur dioxide emissions. Regression (2) shows that the estimated coefficient of local government debt on the number of green invention patent applications per capita is positive at the 1% significance level. In regression (3), the regression coefficient between the number of green invention patent applications per capita and sulfur dioxide emissions is negative at the 5% level. Local government borrowing curbs urban sulfur dioxide emissions by promoting green inventions and innovations, and its intermediary effect is -0.0034, accounting for approximately 14.13% of the total effect. In addition, we also conduct a Sobel test on the above mediation mechanism and find that local government borrowing can promote urban emission reduction by promoting green inventions and innovations. After controlling for the intermediary effect of green invention patents, the regression coefficient of the government debt scale in regression (3) is still negative at the 5% significance level, which suggests that the number of invention patent applications per capita is a partial mediating variable and that local government debt also affects urban emission reduction through other mechanisms.

**Table 7:** Mediation effect analysis.

intermediary variables	(1)	(2)	(3)	(4)	(5)	(6)
	Green invention patent			Green utility model patent		
VARIABLES	*pollution*	*e_inno*	*pollution*	*pollution*	*e_inno*	*pollution*
*debt*	-0.0237***	0.0365***	-0.0204**	-0.0237***	0.0336***	-0.0215**
	(0.0085)	(0.0048)	(0.0086)	(0.0085)	(0.0046)	(0.0086)
*e_inno*			-0.0921**			-0.0671*
			(0.0374)			(0.0392)
control variables	Y	Y	Y	Y	Y	Y
time-fixed effect	Y	Y	Y	Y	Y	Y
individual-fixed effect	Y	Y	Y	Y	Y	Y
*constant*	-3.9927***	-0.3657	-4.0264***	-3.9927***	-0.5935*	-4.0325***
	(0.5944)	(0.3349)	(0.5939)	(0.5944)	(0.3201)	(0.5946)
Observations	2537			2537		
Sobel test	-0.0034(z = -2.341, p = 0.0192)			-0.0023(z = -1.668, p = 0.0954)		
Ratio of indirect effect	14.13%			9.48%		
R-squared	0.8701	0.8368	0.8704	0.8701	0.8392	0.8702

Note:

*** p<0.01,

** p<0.05,

* p<0.1, Standard errors in parentheses.

Regression (4) to regression (6) is the estimation result of the number of green utility model patent applications per capita as the proxy variable of environmental innovation. In regression (4), the estimated coefficient of government borrowing on urban sulfur dioxide emissions is significantly negative, which is consistent with the above conclusion. In regression (5), the regression coefficient of the government debt scale is positive at the 1% significance level, indicating that local government debt effectively promotes the growth of green utility model patents. Regression (6) shows that the regression coefficients of per capita green utility model patents on urban sulfur dioxide emissions are negative at the significance level of 10%, indicating that green utility model innovation can effectively restrain urban sulfur dioxide emissions. Local government borrowing will promote the innovation of urban green utility models and thus have a significant inhibitory effect on urban sulfur dioxide emissions. Its intermediary effect is -0.0023, accounting for approximately 9.48% of the total effect. The Sobel test also confirmed the existence of the intermediary effect of green utility model innovation. In addition, regression (6) shows that after controlling for the intermediary effect of green utility model patents, the estimated coefficient of government debt on urban sulfur dioxide emissions is still negative at the 5% significance level; thus, the number of green utility model patents is also a partial mediating variable.

Based on the above analysis, local government borrowing will promote urban emission reduction by promoting the level of urban environmental innovation, which proves hypothesis 4. Moreover, as two mechanisms through which government borrowing promotes urban emission reduction, the intermediary effect of green invention patents is stronger than that of green utility model patents.

## Discussion

Based on the panel data of 273 cities in China, we conduct a special study on the impact of local government debt on urban environmental pollution and its mechanism. The results show that local government debt can significantly promote the reduction of pollutant emissions, which is consistent with the research results of Carratù et al. (2019) [17]. This conclusion not only affirms the important role of local governments in environmental governance but also has important practical enlightenment for local governments to borrow moderately to reduce environmental pollution. We also find that with the aggravation of urban pollution, the emission reduction effect of government debt shows a dynamic change characteristic of first increasing and then weakening. This conclusion is an important breakthrough in this area of research because the existing literature lacks research on the dynamic characteristics of the impact of local government debt on environmental pollution. In addition, our study also shows that local government debt can stably promote urban emission reduction, and this impact does not show the "U-shaped" characteristics as revealed by Clootens (2017) [15]. Clootens (2017) constructed an overlapping generation model and theoretically deduced that local government debt has the characteristics of first inhibiting and then promoting environmental pollution. On the contrary, based on the urban panel data of China, the largest developing country in the world, we use an empirical test and come to the opposite conclusion. A possible reason for this discrepancy is that compared with developed countries, the debt level of the Chinese government has always been low and has not reached the turning point of a "U-shaped" curve. Moreover, China is a centralized country. For a long time, the central government has strictly limited the scale of local government debt, which makes it difficult for the impact of local government debt on environmental pollution to reach the turning point revealed by Clootens (2017).

We also explore the spatiotemporal heterogeneity of the impact of government debt on urban pollutant emissions based on a subsample regression, which is another supplement to the existing literature. The results show that after the establishment of the Ministry of Environmental Protection in 2008, the role of local government debt in promoting urban emission reduction is more significant. Compared with eastern China, the ecological environment in the central and western regions is relatively fragile, the self-purification ability of the environment is low, and the environmental pollution is relatively serious. Local government debt can more effectively promote urban emission reduction. Therefore, when the subjective will of the government to control environmental pollution is stronger and the external pressure is greater, the promotion effect of government debt on pollutant emission reduction is more significant. This finding expands the research on the impact of government debt on environmental pollution under different systems and regional differences.

Although a few studies have examined the impact of government debt on environmental pollution, they have not deeply investigated its internal mechanism. It is important to clarify the impact of government debt on environmental pollution, but only by deeply exploring its impact mechanism can we provide a basis for deepening theoretical research and guiding policy practice. Therefore, we further study the impact mechanism of government debt on urban emission reduction to compensate for the inadequacy of the existing literature. We find that local government debt can promote urban environmental innovation, which, in turn, contributes to urban emission reduction. This conclusion answers the question regarding why local government debt can affect environmental pollution and provides a new perspective and idea for us to understand the green attribute of government debt.

## Conclusion and insights

Because the ecological environment has the characteristics of an externality and public goods attributes, ecological governance and environmental protection are inherently dependent on government power. Public debt is an important way for the government to raise funds for economic development and will have a significant impact on the ecological environment. Based on panel data from 273 cities in China, this paper uses urban SO_2_ emissions as an example to examine the impact of local government debt on urban emissions reduction and the mechanism through which it operates. The main findings of this paper are as follows. First, local government debt can effectively promote urban emission reduction. Overall, when the urban pollution level is low, local government debt has no significant effect on urban emission reduction, but with an increase in the urban pollution level, the emission reduction effect of government debt presents an inverted "V" dynamic change feature that first increases and then decreases. Second, the impact of local government debt on urban emission reduction is characterized by temporal and spatial heterogeneity. From the perspective of temporal heterogeneity, China established the Ministry of Environmental Protection in 2008. Since then, the scale of local government debt has expanded rapidly, and local government debt plays a significant role in promoting urban emission reduction. However, the emission reduction effect of local government debt was not significant before 2008. From the perspective of spatial heterogeneity, local government debt can effectively promote urban emission reduction in the central and western regions, where the ecological environment is fragile and economic development is relatively lagging. In the eastern region, where the ecological environment is better and the level of economic development is higher, the emission reduction effect of local government debt is not significant. Third, the analysis of the impact mechanism shows that local government debt can promote the level of urban environmental innovation and then promote urban emission reduction, and the intermediary role is stronger for green invention patents than for green utility model patents.

Based on the above conclusions, this paper draws the following policy implications. First, under the premise of reasonably controlling local debt risk, local governments should be appropriately given greater authority over debt, and a financial system that matches the rights of financial expenditure with the responsibility for that expenditure should be established to ensure the local governments’ function in the development of the green economy. In fact, the Chinese government has already launched pilot work on local government debt in an orderly manner, which is helping to strengthen the environmental governance functions of local governments and promote sustainable urban development. Second, it is necessary to fully consider regional differences and adopt diversified measures based on the current status of green economic development in the focal regions to strengthen the role of government debt in promoting urban emissions reduction. In the central and western regions where economic development is relatively lagging and the ecological environment is relatively fragile, as well as in areas where the scale of government debt is relatively small, it is particularly necessary to regulate government debt financing behavior, consider the economic and ecological benefits of government debt, and improve the use efficiency of debt by establishing accountability mechanisms and other incentive and restraint mechanisms. Third, environmental innovation is an important mechanism through which government debt promotes urban emission reduction, but environmental innovation has the attribute of a public good. Therefore, the government should increase support and guidance for environmental innovation, optimize the expenditure structure of government debt, and invest more funds from debt in environmental innovation activities that can balance economic and ecological benefits.

This paper affirms the emission reduction effect of local government debt and thus has important theoretical and practical significance. However, for the environmental effects of local government debt, there are still some problems not investigated in this paper that need to be further studied. First, this paper analyzes the emission reduction effect of government debt through urban panel data, but for data reasons, it lacks a discussion of the micro-mechanism. The formation of macroeconomic phenomena inevitably has a micro-foundation. This paper does not have a sufficient discussion on how local government debt financing can promote emission reduction from the perspective of enterprises or individuals. Exploring the emission reduction effect of government debt from the micro level is an important issue that needs to be resolved in the future. Second, this paper believes that government debt can promote urban emissions reduction by promoting environmental innovation, but other mechanisms have not been analyzed in more detail, and government debt may also indirectly promote urban emission reduction through other mechanisms. For example, government debt is an important financial means for local governments to independently adjust industrial structure, and local government debt may promote urban emission reduction by promoting industrial transformation and upgrading. A comprehensive exploration of the mechanism through which local government debt promotes urban emission reduction is a key issue that needs to be considered in follow-up research.
